# Comparison between Fractionated Dose and Single Dose of Cu-64 Trastuzumab Therapy in the NCI-N87 Gastric Cancer Mouse Model

**DOI:** 10.3390/ijms20194708

**Published:** 2019-09-23

**Authors:** Javeria Zaheer, Hyeongi Kim, Yong-Jin Lee, Sang Moo Lim, Jin Su Kim

**Affiliations:** 1 Division of RI Application, Korea Institute of Radiological and Medical Sciences (KIRAMS), 75 Nowon-ro, Nowon-gu, Seoul 01812, Korea; javeria24@kirams.re.kr (J.Z.); erwin.hyeongi@gmail.com (H.K.); yjlee@kirams.re.kr (Y.-J.L.); smlim328@kirams.re.kr (S.M.L.); 2 Radiological and Medico-Oncological Sciences, University of Science and Technology (UST), 75 Nowon-ro, Nowon-gu, Seoul 01812, Korea

**Keywords:** radioimmunotherapy, single dose (SD), fractional dose (FD), penetration, therapeutics, Herceptin, trastuzumab, gastric cancer

## Abstract

For optimum radioimmunotherapy (RIT), deep penetration and uniform distribution into the tumor core is important. The solid tumor microenvironment, consisting of a highly fibrotic or desmoplastic tumor, abnormal tumor vasculature, high fluid pressure, and the absence of fluid lymphatics, limits the distribution of monoclonal antibodies mAbs to the tumor core. To investigate the optimal rationale for therapeutic mAbs administration and the microdistribution of mAbs, single and serial fractional dosage regimens of Cu-64-trastuzumab (TRZ) with paclitaxel were evaluated. Groups of nude mice were inoculated with gastric cancer cell line NCI-N87 tumor cells. When the tumor size reached 200 ± 20 mm^3^, the mice were divided into two groups for injection of Alexa-647-TRZ. One group (*n* = 5) was injected with 15 mg/kg in a single dose (SD), and the other group (*n* = 5) with two doses of 7.5 mg/kg (fractionated dose (FD)). In both cases, the injections were done intravenously in combination with intraperitoneal paclitaxel either as a SD of 70 mg/kg or fractionated into two doses of 40 and 30 mg/kg. Tumors were harvested, flash frozen, and sectioned (8 µm) five days after Alexa-647-TRZ injection. Rhodamine lectin (rhodamine-labeled *Ricinus communis* agglutinin I, 1 mg in 0.2 mL of phosphate-buffered saline (PBS)) was intravenously injected to delineate the functional vessel for a wait time of 5 min before animal euthanization. Microscopic images were acquired with an IN Cell Analyzer. The amount of TRZ that penetrated the tumor surface and the tumor vessel was calculated by area under the curve (AUC) analysis. For RIT efficacy (*n* = 21), Cu-64-TRZ was injected following the same dose schedule to observe tumor volume and survival ratio for 30 days. The SD and FD regimens of Alexa-647-TRZ were observed to have no significant difference in penetration of mAbs from the tumor edge and vessel, nor was the total accumulation across the whole tumor tissue significantly different. Additionally, the SD and FD regimens of Cu-64-TRZ were not proven to be significantly efficacious. Our study reveals that SD and FD in a treatment design with Cu-64-TRZ and paclitaxel shows no significant difference in therapeutic efficacy on tumor growth inhibition in vivo in mice bearing human gastric cancer xenografts overexpressing HER2 antigen.

## 1. Introduction

With advancements in radioimmunotherapy (RIT), the use of monoclonal antibodies (mAbs) has a clear role in cancer therapy. RIT using mAbs labeled with radionuclides is effective for cancer treatment, because tumor-associated mAbs with cytotoxic radionuclides can selectively bind to tumor antigens [1,2]. RIT drugs such as tositumomab (Bexxar), ibritumomab tiuxetan (Zevalin), and rituximab (Rituxan) have been widely used for the treatment of hematologic tumors, but solid tumors were less responsive to RIT due to insufficient dose delivery and radiation resistance [3]. The tumor microenvironment (TME) of solid tumor limits drug targeting, which results in an insufficient dose delivery of mAb. The factors responsible for limiting the drug delivery in the TME are abnormal tumor vessel structure, extremely fibrotic or desmoplastic tumor, absence of functional lymphatics, and higher fluid permeability [4,5,6,7]. Previously, a number of solutions, such as the use of fractionated doses (FDs) [8], mAb pretargeting [9], and modified targeting agents such as an affibody [10] and recombinant immunotoxin [11], were introduced to improve the effectiveness of RIT. Individual cell survival across the tumor is highly manifested by nonuniform microdistribution of mAbs [12]. 

**Figure 1:**
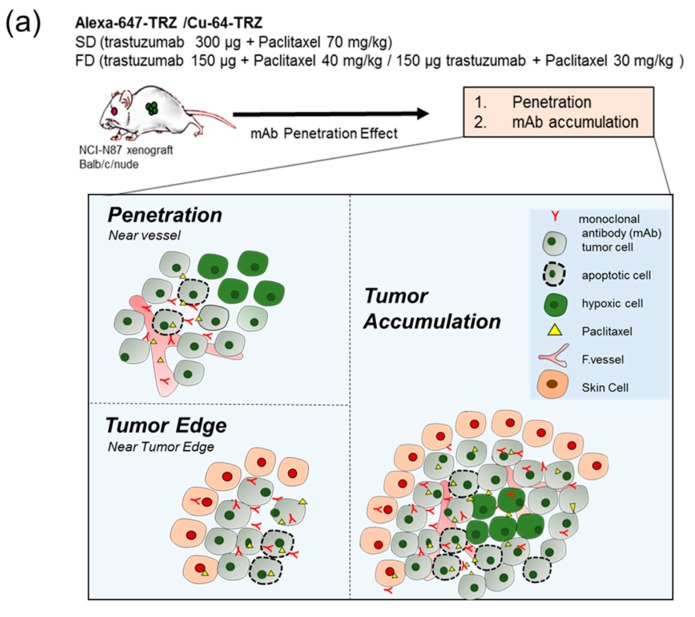

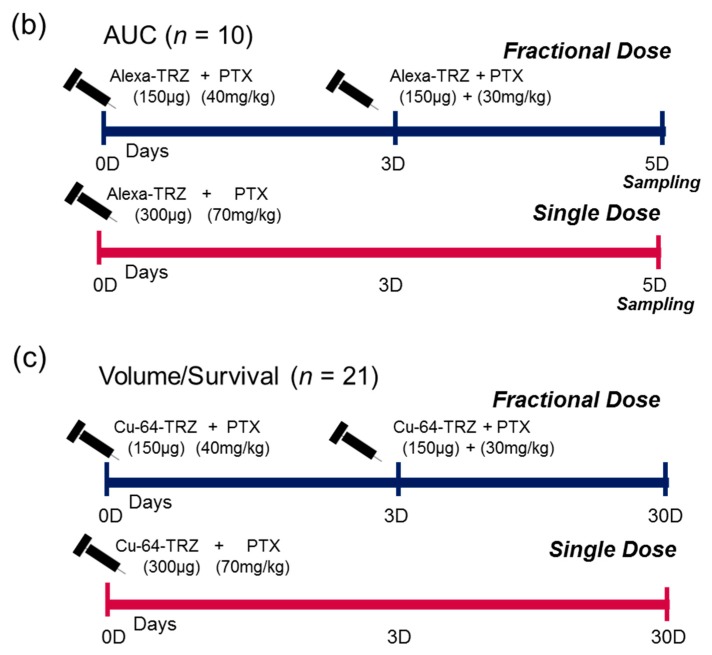
Schematic design of combination therapy by evaluating drug delivery and efficacy. (**a**) Single dose (SD) and fractionated dose (FD) of combination therapy were injected into NCI-N87 xenograft-bearing mice, and penetration of mAb was evaluated from the vessel, near the edge, and total accumulation across the tumor. (**b**) Experimental schedule for Alexa-647-congugated TRZ and Cu-64-labeled-TRZ. (**c**) SD and FD dosage regimens. Cu-64-TRZ, Cu-64-Trastuzumab; Alexa-647-TRZ, Alexa-647-Trastuzumab; PTX, paclitaxel. TRX, trastuzumab.

Since radionuclide sources emit radiation from all directions, while targeting a solid tumor, the surrounding healthy tissue within the radiation range is prone to be harmed by radiation cytotoxicity. Conversely, tumor cells lying outside the range of radiation would escape therapy. Therefore, deeper and uniform distribution is of prime importance in designing RIT. Preliminarily, one of the authors investigated the effects of different doses to quantify mAbs microdistribution in solid tumor tissue, and our analysis of single dose (SD) of B3 (anti-Le^y^ mAb) and fractionated doses of paclitaxel showed promising results, with deeper penetration and uniform distribution of B3, as shown in Appendix A and Supplementary Figure S1. The final results of a previous study showed that total accumulation of B3 per tumor area was not significantly different between serial FD and SD. However, that study was performed using fluorescent-conjugated mAb B3, not radioisotope-labeled mAb B3. High accumulation of radionuclides at the tumor periphery would be more harmful to normal healthy tissues, especially in the case of long-range beta-emitting radioisotopes like Cu-64, Y-90, and I-131. Cu-64, with positron energy of 0.656 MeV and position range of 0.70 mm, also emits β^–^ and Auger electrons and has potential for radiotherapy [13]. Since uniform distribution and deeper penetration of mAbs in tumor tissue are attractive goals in developing RIT for solid tumors, two dosage regimens of Cu-64-trastuzumab (TRZ) with paclitaxel as SD (300 μg TRZ on D0, 70 mg/kg paclitaxel on D1) and FD (150 μg TRZ on D0 or D3, 40 and 30 mg/kg paclitaxel on D1 or D4) could suggest dosage rationalization in terms of their therapeutic efficacy based on our previous results [14]. In this study, we compared the therapeutic efficacy of Cu-64-TRZ RIT between SD and FD in an NCI-N87 xenograft-bearing mouse model. To the best of our knowledge, this is the first report comparing mAb microdistribution and RIT efficacy using the therapeutic antibody TRZ. Figure 1a shows a graphical representation and Figure 1b,c shows the experimental timeline.

## 2. Results

The microdistribution of TRZ in tumor tissue was evaluated. The microdistribution was measured from the tumor edge and from the functional vessel surrounding the tumor tissue. The results of the study are described in the following subsections.

### 2.1. Alexa-647-Trastuzumab Penetration from the Tumor Vessel

**Figure 2:**
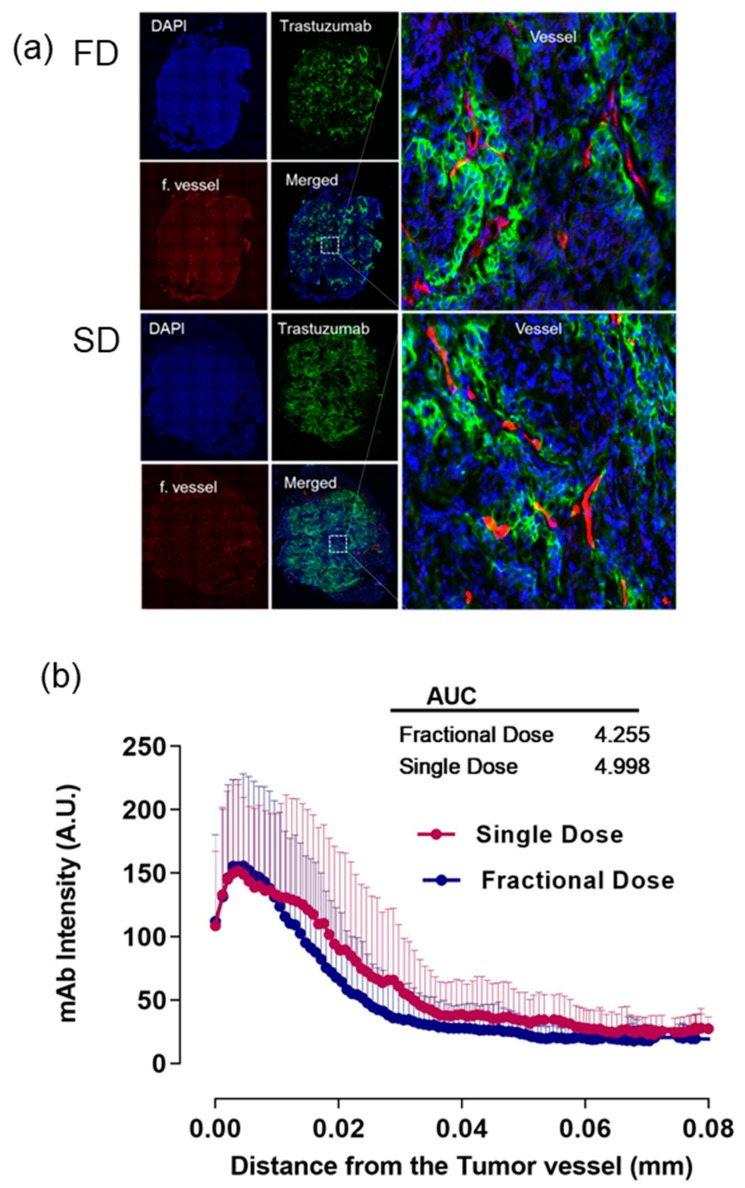
Representative images of SD and FD. (**a**) DAPI (4′,6-diamidino-2-phenylindole) in blue, TRZ (trastuzumab) intensity in green, functional vessel in red, merged images, and zoomed merged area near vessel. (**b**) Histogram plot shows line profiling comprising mean and standard deviation as error bars (above) of SD (single dose) and FD (fractional dose) with respective AUC values.

Alexa-647-TRZ penetration from the vessels in tumor tissue sections was calculated by drawing volume of interest (VOI) lines from the tumor functional vessels. Figure 2a shows representative images of FD and SD displaying DAPI (4′,6-diamidino-2-phenylindole) in blue, TRZ intensity in green, functional vessel in red, merged images, and zoomed merged area near vessel. Line profile data were prepared from various vessels observed in the tumor tissue section surrounding the periphery and center of the tissue and analyzed by area under the curve (AUC) analysis. In terms of the penetration depth, the AUC value of Alexa-647-TRZ intensity in SD is 4.998, whereas for FD, the value is 4.255, implying no statistical difference between the groups. Figure 2b shows histogram data plotted as a line graph.

### 2.2. Alexa-647-Trastuzumab Penetration near the Tumor Edge

**Figure 3:**
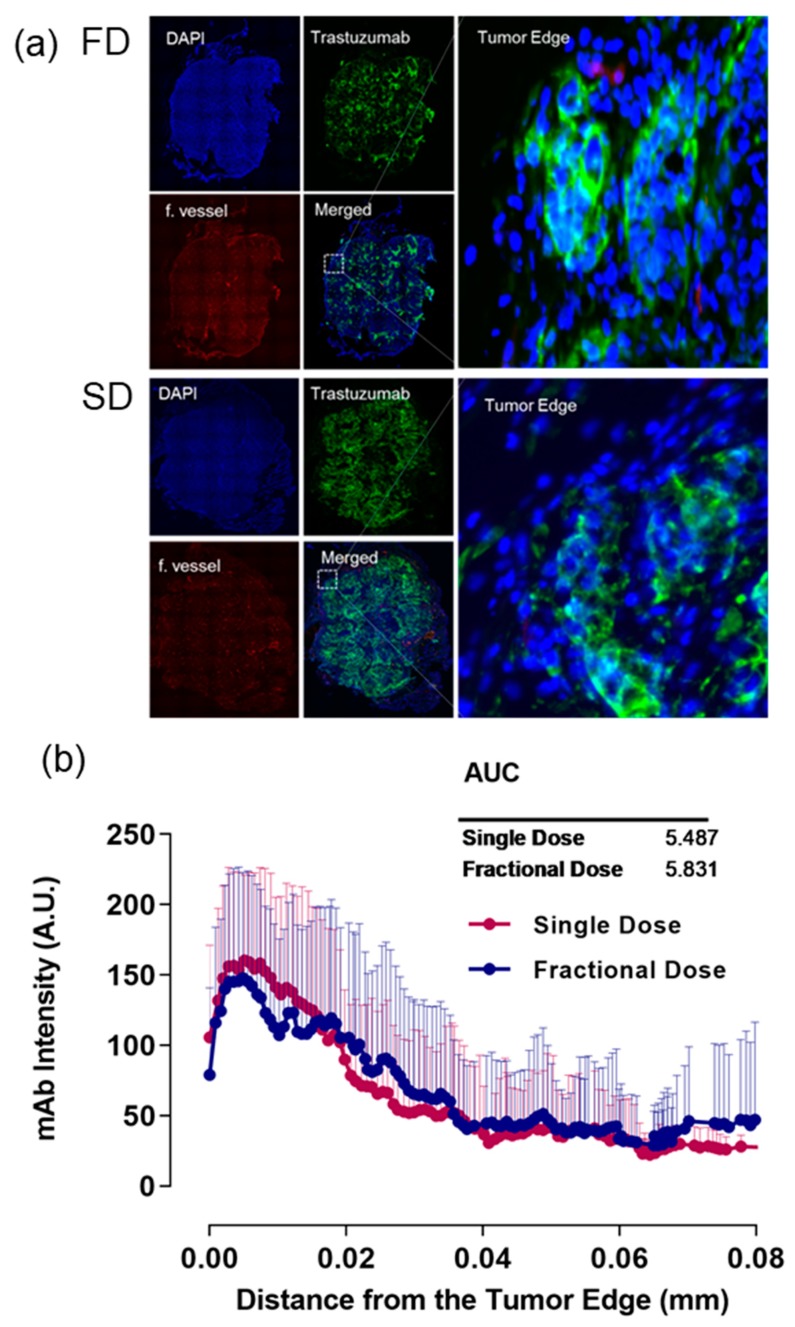
Representative images of SD (single dose) and FD (fractional dose). (**a**) DAPI in blue, TRZ (trastuzumab) intensity in green, functional vessel in red, merged images, and zoomed merged area near tumor edge. (**b**) Histogram plot shows line profiling comprising mean and standard deviation as error bars (above) of SD and FD with respective AUC values.

Alexa-647-TRZ penetration near the edge in tumor tissue sections was calculated by drawing VOI lines near the edge toward the center. Figure 3a shows representative images of FD and SD displaying DAPI in blue, TRZ intensity in green, functional vessel in red, merged images, and zoomed merged area near the tumor edge. Line profile data were generated from the tumor edge toward the center and analyzed by AUC analysis. The AUC for SD near the edge is 5.487 and for FD is 5.831. There is no significant difference between FD and SD in TRZ intensity near the tumor surface. Figure 3b shows histogram data plotted as a line graph.

### 2.3. Alexa-647-Trastuzumab Accumulation across Whole Tumor

**Figure 4:**
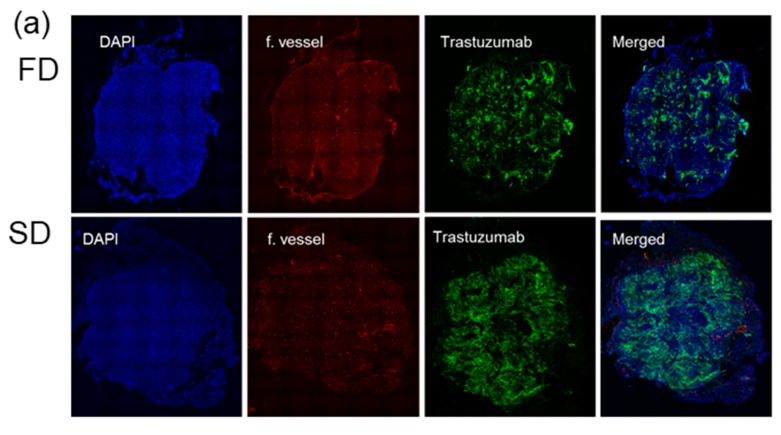

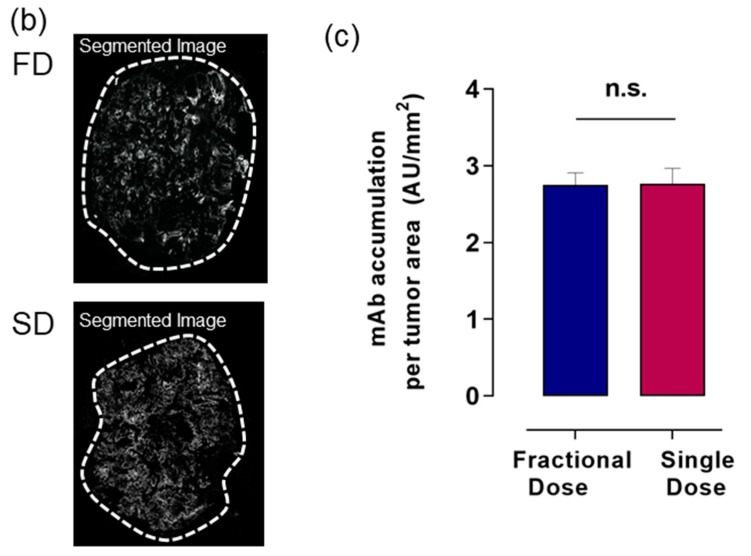
Representative images of SD (single dose) and FD (fractional dose). (**a**) DAPI in blue, TRZ (trastuzumab) intensity in green, functional vessel in red, merged images showing accumulation of TRZ across the whole tumor. (**b**) Segmented TRZ image and polyline traced across the tumor surface. For intensity quantification, colored images from ZEN blue were traced with polyline, and (**c**) mAb accumulation per tumor area was plotted as a bar graph. Data shown as means and standard deviation as error bar. n.s., nonsignificant.

Next, we validated the difference between the two groups in total TRZ accumulation by measuring the TRZ intensity across the whole tumor tissue image. Tagged image file format (TIFF) images of TRZ generated from Zen (blue) software (Carl-Zeiss Microscopy, Oberkochen, Germany) was exported to the MIPAV program (National Institutes of Health, Bethesda, MD, USA). A polyline VOI tracing the tumor surface and calculating the total signal intensity was obtained from the image. A comparison of the SD of 300 µg of TRZ plus 70 mg/kg of paclitaxel on D0 and D1 with the FD of 150 µg of TRZ and 40 mg/kg of paclitaxel on D0 and D1 and 150 µg of TRZ and 30 mg/kg of paclitaxel on D3 and D4 shows no significant difference in TRZ accumulation (total mAb intensity in the tumor/tumor area) between FD and SD. Figure 4a shows representative images of FD and SD; Figure 4b shows the segmented TRZ image and polyline traced across the tumor surface. For intensity quantification, colored images from ZEN blue were traced with polyline, and Figure 4c shows mAb accumulation per tumor area plotted as a bar graph.

### 2.4. Cu-64-Labeled Trastuzumab Efficacy in Tumor Size Reduction

**Figure 5:**
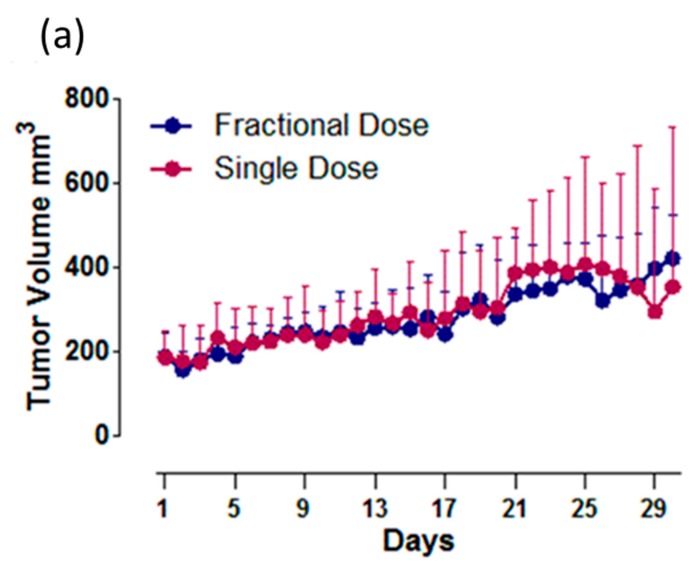

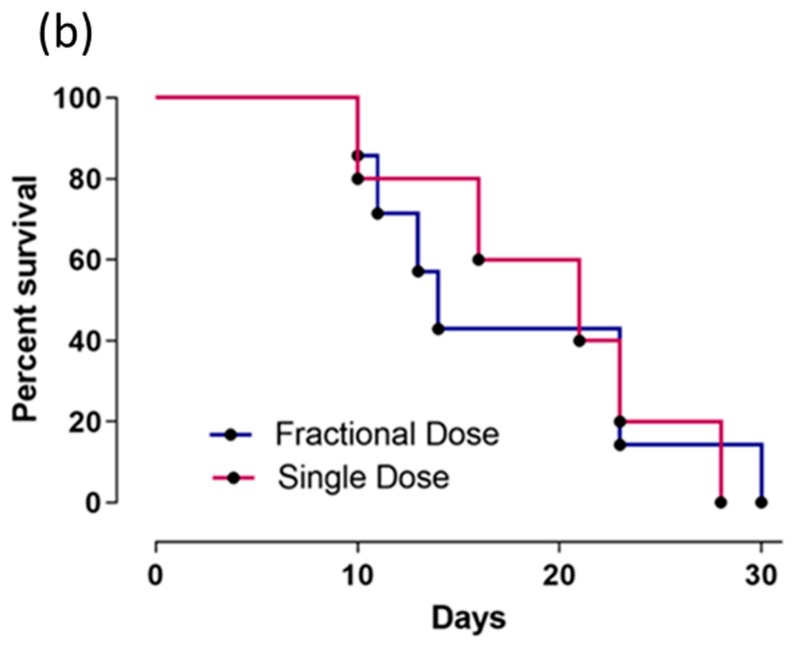
(**a**) Plot of tumor data showing means of tumor volume for each group with standard deviations as error bars (above). (**b**) Kaplan–Meier curves for survival data of FD (fractional dose) and SD (single dose) were recorded up to 30 days. The data are statistically nonsignificant.

The efficacy of RIT of Cu-64-TRZ was evaluated in a tumor volume reduction and survival study. The two groups of SD (354 µCi/300 µg on D0, 70 mg/kg paclitaxel on D1) and FD (168 µCi/150 µg on D0, 40 mg/kg on D1 and 165 µCi/150 µg on D3, 30 mg/kg paclitaxel on D4) were evaluated on the NCI-N87 bearing xenograft mice. Tumor volumes were recorded for a month and survival data were plotted on a Kaplan–Meier plot. Tumor volume and survival curves are shown in Figure 5a,b. There was no significant difference in tumor volume reduction between the groups.

## 3. Discussion

In the present study, to overcome binding site barriers and improve the therapeutic effectiveness of radiolabeled mAbs, the effects of different doses, as SD or FD, of Alexa 647-TRZ and paclitaxel on their accumulation and microdistribution were investigated. For this purpose, the microdistribution of Alexa 647-TRZ inside the tumor tissue was assessed.

During RIT, deep penetration and uniform distribution of beta particle–emitting radioisotope-labeled mAbs in the tumor microenvironment is important to protect normal tissue [15]. Previous studies showed that most mAbs remains distributed in the tumor periphery rather than the core [16,17,18]. The results of previous studies have shown that the accumulation of mAbs is enhanced after treatment with paclitaxel (Taxol) [17] or pulsed high-intensity focused ultrasound during RIT for solid tumors [17,19]. Although the accumulation of mAbs was enhanced using these approaches, its distribution was still restricted to <25–50 μm from the surface of the tumor during combination therapy using physiological delivery promoters, such as bevacizumab (Avastin) for antiangiogenic treatment [20] and paclitaxel (Taxol) [17], and physical modifiers, such as pulsed high-intensity focused ultrasound [17,19]. 

We evaluated the combination treatment in our present study with the therapeutic antibody TRZ, which binds to the overexpressed HER2 antigen in NCI-N87 xenograft as SD and FD. We aimed to determine whether reducing interstitial pressure could overcome heterogeneous binding of mAb-antigen by uniform distribution. Elevated interstitial pressure that limits the inward transport of mAbs from the periphery is assumed to be reduced by the paclitaxel cytotoxic effect. When higher doses of TRZ combined with paclitaxel were tested in our study as SD and FD, we obtained nearly similar patterns of antibody penetration in both groups as analyzed by tumor edge, periphery, vessel, and total tumor accumulation. A study by Joseph et al. compared fractionated RIT vs. single dose by mathematical modelling and proposed that for a heterogeneous absorbed dose, rapid fractionation of RIT has a therapeutic advantage, and for a homogeneous absorbed dose, a large single dose of RIT would be better for tumor cure. However, expected remission was predicted to be similar for both models, i.e., fractionated and single treatment [21]. RIT labeled with beta-emitting particles such as Y-90, Lu-177, or Cu-64 could be efficacious in this dosage regimen, because high accumulation of RIT toward the periphery could be dangerous for healthy tissue. Therefore, in the present study we compared the two groups with TRZ in the NCI-N87 xenograft mouse model and investigated whether Cu-64-labeled TRZ could be beneficial in either of the two dosage regimens. However, the result did not show a significant difference in TRZ intensity between the groups or that the efficacy of RIT was superior or inferior. The potential advantage of the SD scheme is that it can tolerate less sublethal damage that can be repaired with bone marrow transplantation, while FD promotes more uniform distribution and reduced toxicity, depending upon the tumor type [22]. Many studies report that fractionated doses produce better responses [23]. Therefore, the rationale in dose adjustment is important to obtain the optimum therapeutic response by dose fractionation.

Cu-64-TRZ could be used for both diagnosis and therapy. Previously, an antigen-responsive molecular sensor study with TRZ [24], a comparison study of chelator [25], dosimetry studies of Cu-64-TRZ PET [13,26], and a study of I-131 TRZ using a HER2+ NCI N87 xenograft mouse model [27] were conducted. However, tumor microenvironments such as angiogenesis, fibrosis, and proliferation were not considered in those previous studies [13,24,25,26,27].

In this study, we compared SD and FD of Cu-64-TRZ after TME modulation using paclitaxel. Previously, Cu-64-TRZ for therapy of brain metastases in HER2-positive breast cancer was introduced [28]. One possible application for further study would be a comparison of SD or FD of Cu-64-TRZ for brain metastasis in HER2-positive breast cancer.

## 4. Materials and Methods

### 4.1. Cell Culture

NCI-N87 HER2-positive gastric cancer cell line was obtained from American Type Culture Collection (ATCC), and maintained in Roswell Park Memorial Institute (RPMI) containing 10% fetal bovine serum with 5% antibiotics (Sigma, St. Louis, MO, USA) at 37 °C in a humidified 5% CO_2_ incubator. NCI-N87 cells (5 × 10^6^) were subcutaneously injected into male BALB/nude mice. Tumor size was measured using a digital caliper, and the volume was calculated by the formula: width^2^ × length × 0.5.

### 4.2. Animal Model and Treatment Plan

All mouse-related experiments were performed under a protocol approved by Institutional Animal Care and Use Committee (IACUC) (number KIRAMS 2018-0016; date of approval: 15 May 2018) of the Korea Institute of Radiological and Medical Sciences (KIRAMS). Animal were 5 to 6 weeks old and weighed approximately 18–20 g. The treatment plan consisted of 2 groups, SD and FD. SD was formulated by conjugation of Alexa-647-TRZ (300 μg TRZ on D0 and 70 mg/kg of paclitaxel on D1), and FD consisted of dose fractionation of Alexa-647-TRZ (150 μg TRZ on D0 and D3, paclitaxel 40 mg/kg on D1, and 30 mg/kg on D4). Upon reaching 200 ± 20 mm^3^ tumor volume, the animals were re-caged and grouped according to the treatment plan. The survival study followed the same dosage schedule with Cu-64-labeled TRZ.

### 4.3. Conjugation of Alexa Fluor 647 to Trastuzumab

A solution of Alexa-647-NHS ester (Invitrogen, Waltham, MA, USA) was prepared in dimethyl sulfoxide (DMSO) containing 1% acetic acid and dissolved in 500 μL of TRZ (10 mg/mL) in 1 M sodium bicarbonate solution with pH 8.4. The reaction was incubated for 1 h at room temperature. After 1 h, the reaction was purified by a size exclusion PD-10 column (GE Healthcare Bio-Sciences AB, Uppsala, Sweden) connected with an ultraviolet/visible detector set at a maximum wavelength of 517 nm. An aliquot (100 μg/μL, PBS, pH 7.2) of unconjugated and Alexa 647-antibody conjugate was measured with a NanoDrop spectrophotometer (Thermo Fisher Scientific, Waltham, MA, USA). The number of Alexa 647 molecules surrounding TRZ was estimated by comparing the peak intensity between conjugated Alexa 647-TRZ and Alexa 647 free eluted solution on a high-performance liquid chromatography profile. The concentration of Alexa 647-TRZ was calculated and adjusted per dose.

### 4.4. Labelling of Cu-64-Trastuzumab

We used 50 MeV cyclotron irradiation to produce Cu-64 at KIRAMS. Monoclonal antibody TRZ (20 mg) (Herceptin; F. Hofmann–La Roche, Basel, Switzerland) was chelated with 1,4,7,10-tetraazacyclododecane-1,4,7,10-tetraacetic acid (DOTA) by adding it to the DOTA-NHS-ester (1 mg, macrocyclic) in 800 μL of 1 M sodium bicarbonate buffer (pH 8.5). The mixture was gently mixed for 24 h at 4 °C. Then the solution was filtered using a PD-10 column with 1 mM sodium acetate (pH 5.5) to remove the unconjugated chelator. Labelling of Cu-64-DOTA-TRZ was performed by direct conjugation as ^64^CuCl2 (370 MBq) was added to 1 mg of DOTA–TRZ in 1 mM sodium acetate (pH 5.5). The reaction mixture was incubated for 1 h at 37 °C. The percent yield of radiolabeling was confirmed by instant thin-layer chromatography on silica gel (solvent: citric acid). The obtained yield was ≥97%. Cu-64-labeled TRZ was delivered in the same manner to both FD and SD, and tumor size and survival rate were recorded for a month. The immunoreactivity of Cu-64-DOTA-TRZ was determined to be 92.8% using a cell-binding assay and the specific activity was 4.93 ± 0.87 mCi/mg.

### 4.5. Immunofluorescence Staining

The harvested tumors were fixed with 4% paraformaldehyde overnight and then cryopreserved with 30% sucrose solution until the tumor tissue sank to the bottom of the centrifuge tube. Subsequently, tumor tissues were embedded in optimum cutting temperature compound (OCT) and frozen at –70 °C until use. The frozen embedded tissues were sectioned to 8 µm thickness using a Leica CM 1850 cryostat (Leica Microsystems, Wetzlar, Germany), rehydrated with PBS, stained with DAPI, and observed using a fluorescent microscope (IN Cell Analyzer 2200, GE Healthcare, Chicago, IL, USA).

### 4.6. Histological Image Acquisition

Fluorescent images were acquired with 10× objective lenses using 3 independent channels. IN Cell Analyzer was customized with fluorescent microscopy and mosaic stitching software (IN Cell Developer Toolbox, GE Healthcare, USA). The channels were as follows: DAPI for nuclei (shown in blue, Ex/Em = 358/461 nm; exposure 0.9), Cy5 channel for Alexa 647-TRZ (shown in green, Ex594/ Em665; exposure 0.3), and Cy3 channel for rhodamine lectin to detect blood vessels (shown in red, Ex550/Em575; exposure 0.8). Offset was determined by Autofocus.

### 4.7. Penetration and Accumulation of Alexa-647-Trastuzumab

Image analysis was performed using an in-house program written in MATLAB (MathWorks, Natick, MA, USA), ZEN (blue), and MIPAV (National Institutes of Health, Bethesda, MD, USA). Individual channels were exported from ZEN (blue) in TIFF files for further processing in MIPAV.

TRZ intensity from the edge of the tumor was calculated by drawing volume of interest (VOI) lines perpendicular from the edge toward the center. The resulting graph depicting line intensity vs. line distance was read in MATLAB with the in-house program. The TRZ signal intensity was measured corresponding to 80 μm line distance. The penetration of TRZ from the tumor edge was calculated using the AUC analysis from 3 tumors with 10 sections in each tumor (*n* = 30) in 2 groups. The mAb penetration from tumor vessels was similarly calculated with line profiling by measuring the intensity from the vessel in various regions of interest (ROIs) from peripheral and central regions in each tumor. In addition, the total Alexa 647-TRZ accumulation was calculated as follows:

Total tumor intensity of mAb = intensity of mAb in the tumor/total tumor area.

### 4.8. Statistical Analysis

Data are expressed as mean ± standard deviation. Graph Pad Prism 5 (Graph Pad Software, La Jolla, CA, USA) was used for statistical analysis.

## 5. Conclusions

Deeper and more uniform distribution toward the tumor core is important in RIT delivery in solid tumors, not only because of the therapeutic efficacy but also because it limits normal cell damage. Dose optimization as SD and FD shows no significant therapeutic efficacy with a combined RIT of Cu-64-TRZ and paclitaxel in the gastric cancer mouse model.

## Supplementary Materials

The following are available online at https://www.mdpi.com/1422-0067/20/19/4708/s1.

## Appendix A

### Appendix A.1. Tumor Model

Animal experiments were performed under a protocol approved by the National Institutes of Health Animal Care and Use Committee. Tumor xenografts were established by subcutaneous inoculation of 3 × 10^6^ A431 cells in 0.1 mL PBS into the right flank of athymic mice (*n* = 6 per group, 5–6 weeks, 18–20 g; NCI-DCT, Frederick, MD, USA). Tumor dimensions were measured using a caliper. Tumor size (mm^3^) was calculated using the following formula: a × b^2^ × 0.4, where a is the largest and b is the smallest diameter (in mm) of the tumor.

### Appendix A.2. Antibody Penetration Studies

Paclitaxel was used as promoter to enhance the physiological delivery of Alexa mAb B3. Paclitaxel is a plant alkaloid that promotes the stabilization of microtubules, leading to mitotic arrest at the radiosensitive G2/M phase that disrupts cell division and causes cell death [29]. When the tumor size reached ~200 mm^3^, the following experiments were performed to determine the effects of a single mAb dose of 150 µg of Alexa mAb B3 as group 1 (baseline); paclitaxel dose (serial dose of 150 µg Alexa mAb B3 and 40 or 70 mg/kg paclitaxel) as group 2, exp. 1; and serial fractional doses (serial dose of 300 µg Alexa mAb B3 and 70 mg/kg paclitaxel) as group 2, exp. 2. The drug treatment timeline is tabulated in Supplementary Table S1. The paclitaxel doses of 40 and 70 mg/kg used in the present study were equivalent to doses of 123 and 215 mg/m^2^, respectively, in humans, which is within the range of doses typically used to treat women with metastatic breast cancer (100–250 mg/m^2^). Two or five days after the injection of Alexa mAb B3, the mice received a lateral tail vein injection of rhodamine-lectin (rhodamine-labeled *Ricinus communis* agglutinin I, 1 mg in 0.2 mL PBS) to delineate the blood vessels; 5 min after the lectin injection, the mice were euthanized by CO_2_ inhalation and exsanguinated by cardiac puncture before dissection. Tissue imaging was performed with 20× objective (pixel size = 0.464 µm) using a ScanScope FL (Aperio, Vista, CA, USA) equipped with a motorized scanning stage. Image analysis was performed using an in-house program written in MATLAB (MathWorks, Natick, MA, USA). The penetration of Alexa mAb B3 up to ~1 mm depth from the tumor edge was calculated using area under the curve (AUC) analysis. The total accumulation of Alexa mAb B3 was calculated by dividing the total Alexa mAb B3 intensity in the tumor by the total tumor area. Compared with the single dose of 150 μg Alexa mAb B3, 40 and 70 mg/kg paclitaxel produced 1.8-fold (*p* < 0.005) and 2.0-fold (*p* < 0.005) greater penetration of Alexa mAb B3 to 1.0 mm from the tumor surface, respectively, as calculated by the AUC method.
