# Supplementary material for: Comparison between Fractionated Dose and Single Dose of Cu-64 Trastuzumab Therapy in the NCI-N87 Gastric Cancer Mouse Model
Source: Int J Mol Sci. 2019 Sep 23;20(19):4708. doi: 10.3390/ijms20194708 (PMC6801605; doi:10.3390/ijms20194708)
Supplement: Supplementary file 1 [file ijms-20-04708-s001.pdf]

Supplementary Materials

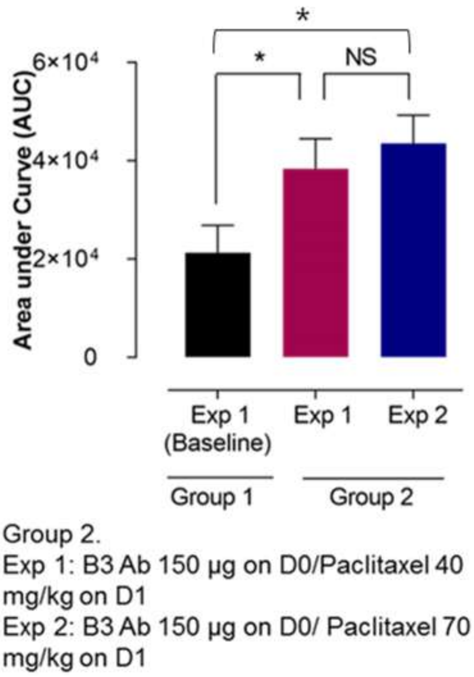

Supplementary Figure S1. Increased dose of paclitaxel improves B3 accumulation. * $p < 0.005$.

Table S1. Dosage scheme.

	Drug	Day 0	Day 1	Day 2	Day 3	Day 4	Day 5
Group 1							
Experiment 1	Alexa mAb B3* (µg)	150					Sacrificed
Group 2							
Experiment 1	Alexa mAb B3 (µg)	150					
	Paclitaxel (mg/kg)		40				Sacrificed
Experiment 2	Alexa mAb B3 (µg)	150					
	Paclitaxel (mg/kg)		70				Sacrificed

* $p < 0.005$.
